# The Small Regulatory RNA Spot42 Inhibits Indole Biosynthesis to Negatively Regulate the Locus of Enterocyte Effacement of Enteropathogenic *Escherichia coli*

**DOI:** 10.3390/microorganisms5040078

**Published:** 2017-12-01

**Authors:** Shantanu Bhatt, Valerie Jenkins, Elisabeth Mason, Sarah Muche

**Affiliations:** 1 Department of Biology, Saint Joseph’s University, Philadelphia, PA 19131, USA; Valerie.Jenkins@som.umaryland.edu (V.J.); emmason@liberty.edu (E.M.); sm644863@sju.edu (S.M.); 2 Department of Chemistry, Saint Joseph’s University, Philadelphia, PA 19131, USA

**Keywords:** posttranscriptional, Spot42, *tnaCAB*, indole, LEE, EPEC

## Abstract

The locus of enterocyte effacement is necessary for enteropathogenic *Escherichia coli* (EPEC) to form attaching and effacing (A/E) lesions. A/E lesions are characterized by intimate bacterial adherence to intestinal cells and destruction of microvilli, which leads to diarrhea. Therefore, studies interrogating the regulation of the locus of enterocyte effacement (LEE) are critical for understanding the molecular epidemiology of EPEC infections and developing interventional strategies. Hitherto, most studies have centered on protein-based regulators, whereas the role of small regulatory RNAs remains underappreciated. Previously, we identified the first sRNAs—MgrR, RyhB, and McaS—that regulate the LEE of EPEC. This study was undertaken to identify additional sRNAs that impact the LEE. Our results suggest that the catabolite-responsive sRNA, Spot42, indirectly controls the LEE by inhibiting synthesis of its inducer, indole. Spot42 base-pairs with the *tnaCAB* mRNA and presumably destabilizes the transcript, thereby preventing expression of the regulatory and structural proteins that are involved in the import and hydrolysis of tryptophan into indole. The absence of intracellular indole leads to reduced transcription of the *LEE1*-encoded master transcriptional activator Ler, thereby maintaining the LEE in its silenced state and delaying A/E lesion morphogenesis. Our results highlight the importance of riboregulators that synchronize metabolic and virulence pathways in bacterial infection.

## 1. Introduction

Small regulatory RNAs (sRNAs) constitute a structurally and mechanistically assorted class of regulatory nucleic acids that confer numerous advantages upon their host bacterium [1,2]. For instance, sRNAs, due to their small size and the fact that they forgo translation, are metabolically inexpensive to synthesize and maintain, thereby shortening the response time for cells to acclimate and adapt to environmental fluctuations [3,4]. sRNAs also enhance the regulatory and phenotypic range by fine-tuning the transcriptional output [1,2,4]. Additionally, sRNA-regulated circuits are more tolerant to mutational events. A subclass of sRNAs, termed trans-encoded sRNAs, are encoded distantly from their regulated targets. These sRNAs share limited complementary to their target mRNAs and, thus, typically require assistance from a chaperone protein to facilitate base-pairing interactions [1]. One of the most frequently used chaperones is the molecular matchmaker Hfq. The holoprotein functions as a homohexameric toroid [5]. Hfq binds to a sRNA at one surface and an mRNA on the other, bringing the sRNA-mRNA pair in proximity to facilitate base-pairing [1,2,6]. Base-pairing of the sRNA to the mRNA can result in a varying range of regulatory outcomes including transcriptional or translational regulation or mRNA processing [1]. Together, Hfq and Hfq-dependent sRNAs control numerous cellular processes such as motility, biofilm formation, response to stressors, and virulence, to name a few [1,6]. In particular, the prominence of Hfq and Hfq-dependent sRNAs in the virulence of diverse, phylogenetically distant, pathogens is well-established [2]. For instance, in *Salmonella enterica* serovar Typhimurium and enterohemorrhagic *Escherichia coli* (EHEC), genome-wide approaches have been undertaken to identify the full complement of sRNAs involved in virulence [7,8,9]. However, for others, such as enteropathogenic *E. coli* (EPEC), the repertoire of Hfq-dependent sRNAs remains undefined.

EPEC belongs to the attaching and effacing (A/E) morphotype and predominantly causes diarrhea amongst infants [10], by intimately adhering to intestinal cells and destroying the microvilli [4,10,11,12]. Intimate bacterial attachment initiates a signal transduction cascade that leads to ultrastructural remodeling of the host cytoskeletal proteins beneath the bacterium to form a membrane-enclosed protrusion from the infected cell, termed “pedestal” or A/E lesion [4,11,13,14,15]. The disintegration of microvilli reduces water and nutrient uptake by intestinal cells, leading to diarrhea.

The locus of enterocyte effacement (LEE) pathogenicity island is necessary and sufficient for EPEC to form A/E lesions [16]. The LEE is complex morphogenetic element that houses the genes for a type 3 secretion system (T3SS) [4,13,17]. The T3SS bridges the bacterium to the host cell and facilitates the smuggling of effector proteins into the host where they subvert host signaling pathways to initiate A/E lesion formation [4,13,18]. The importance of this genomic island to the bacterium is illustrated by the characterization of over 40 regulatory factors that control the LEE [4,19]. Most regulators target one of the two key regulatory operons—*grlRA* (*LEE7*) and/or *LEE1.* The *grlRA* operon encodes the transcriptional antiactivator GrlR and the transcriptional activator GrlA [20], whereas the first gene of the *LEE1* operon specifies the master transcriptional regulator of the LEE, Ler [21]. GrlA binds to and activates transcription from the *LEE1* operon [22], whereas GrlR associates with GrlA and sequesters it, thereby antagonizing its effect [23]. Upon expression, Ler coordinates transcription from other LEE operons leading to pedestal formation [21].

In a previous study, we reported the first, and thus far, only sRNAs—MgrR and RyhB—that directly regulate the LEE in EPEC [19]. The present study was undertaken to identify sRNAs that indirectly regulate the LEE by modulating gene expression from the well-known regulatory operon *tnaCAB*. The *tnaCAB* operon, originally identified and characterized in *E. coli* [24], is conserved in its pathogenic and nonpathogenic lineages [25]. This transcription unit enables its host bacterium to import and metabolize tryptophan as the sole carbon and energy source [26]. *tnaC* encodes a *cis*-acting leader peptide that regulates the expression of the cotranscribed genes, *tnaA* and *tnaB* [27]. *tnaA* encodes the enzyme tryptophanase [28], whereas *tnaB* specifies a tryptophan transporter [29]. Imported tryptophan is subsequently hydrolyzed by tryptophanase into indole, pyruvate, and ammonia [30]. In EPEC, indole induces transcription from the *LEE1* operon and promotes A/E lesion formation [25,31]. The *tnaCAB* mRNA is a hub for posttranscriptional regulation. For instance, in *E. coli*, the *tnaCAB* message is regulated by Rho-dependent transcriptional termination [32], and by tryptophan-dependent antitermination [33]. Recently, two sRNAs, GlmY and GlmZ, were shown to repress *tnaA* in EHEC [9]. We proceeded to test if other sRNAs regulated the *tnaCAB* mRNA, since such candidates would conceivably affect the LEE.

Our results reveal a novel role for the catabolite-responsive sRNA Spot42 as a riboregulator of the *tnaCAB* mRNA and the LEE of EPEC. Our results reveal that Spot42 negatively regulates indole synthesis by base-pairing to the *tnaC-tnaA* intergenic region and repressing the entire *tnaCAB* mRNA. In the absence of indole, transcription of *ler* is reduced. Thus, our results suggest that Spot42 is a novel sRNA regulator of the LEE in EPEC.

## 2. Materials and Methods

### 2.1. Media, Bacterial Strains, Antibiotics, Plasmids, and Primers

Bacteria were routinely propagated in Luria-Bertani (LB) broth or LB agar. The medium was supplemented with appropriate antibiotics and/or inducers when necessary. The following supplements were used at appropriate concentrations: Streptomycin (100 µg/mL), chloramphenicol (12.5–25 µg/mL), kanamycin (50 µg/mL), tetracycline (15 µg/mL), ampicillin (100 µg/mL), Isopropyl β-d-1-thiogalactopyranoside [IPTG] (1 mM), and arabinose (0.02%). Strains, plasmids, and oligonucleotides used are listed in Table 1 and Table 2. For quantitative assays (i.e., indole synthesis, β-galactosidase, qRT-PCR, and Western blotting), cultures were grown at 37 °C/250 rpm to an optical density of 1.1–1.4 and processed as described below.

### 2.2. DNA Manipulations

Polymerase chain reactions (PCRs), restriction digestions, ligations, cloning, and transformations were performed following standard protocols [38]. Chromosomal modifications in *E. coli* were engineered by lambda red-mediated recombineering essentially as described in our previous paper [4].

### 2.3. Screen to Identify Hfq-Dependent sRNA Regulators of Indole Biosynthesis

Electrocompetent EPEC was transformed with individual members of a plasmid library, each of which overproduces a solitary Hfq-dependent sRNA of *E. coli*, under the transcriptional control of an IPTG-inducible promoter. The plasmid library was a generous gift from Susan Gottesman, in whose lab it was engineered [39]. Thereafter, the EPEC transformant library was screened to identify sRNAs that regulate indole biosynthesis. Briefly, an axenic colony of each EPEC transformant was inoculated in LB broth supplemented with ampicillin and cultured overnight at 37 °C under shaking conditions. The next day, each transformant was sub-cultured 100-fold in LB broth supplemented with ampicillin and IPTG and grown to an OD_600_ of 1.2–1.4. One mL aliquot of each culture was assayed for indole biosynthesis by addition of 5 drops of Kovac’s reagent. Kovac’s reagent reacts with indole to produce a red colored dye, rosindole. Rosindole production was visually monitored over a period of 20 s, after which the cultures were photographed.

### 2.4. Beta-Galactosidase Assay

Beta-galactosidase assays on the translational fusion (*P_araBAD_-tnaA’-‘lacZ*) containing reporter strains were performed as described in our previous paper [19], with the exception that bacteria were grown to an optical density of 1.1–1.4 in LB supplemented with ampicillin, arabinose, and IPTG. The same conditions, as described above, were employed for the transcriptional fusion (*P_LEE1_-LEE1’-lacZ^+^*) containing reporter strains with the omission of arabinose from the medium.

### 2.5. RNA Isolation and qRT-PCR

RNA isolation and qRT-PCR were performed as described in our previous paper [19]. The steady-state mRNA levels were normalized with respect to the housekeeping 16S rRNA transcript.

### 2.6. Preparation of Cell Lysates for Western Blotting

Bacterial cultures were processed for western blotting as described previously [19]. The abundance of tryptophanase protein was measured by using a polyclonal anti-tryptophanase antibody, at a 4000-fold dilution, which was purchased from Assaypro (St. Charles, MO. Cat # 33517-05111). Protein loading was controlled for by staining for the total protein transfer using Ponceau S stain.

## 3. Results and Discussion

### 3.1. Overproduction of Spot42 Inhibits Indole Biosynthesis by Down-Regulating the tnaCAB mRNA

**Figure 1:**
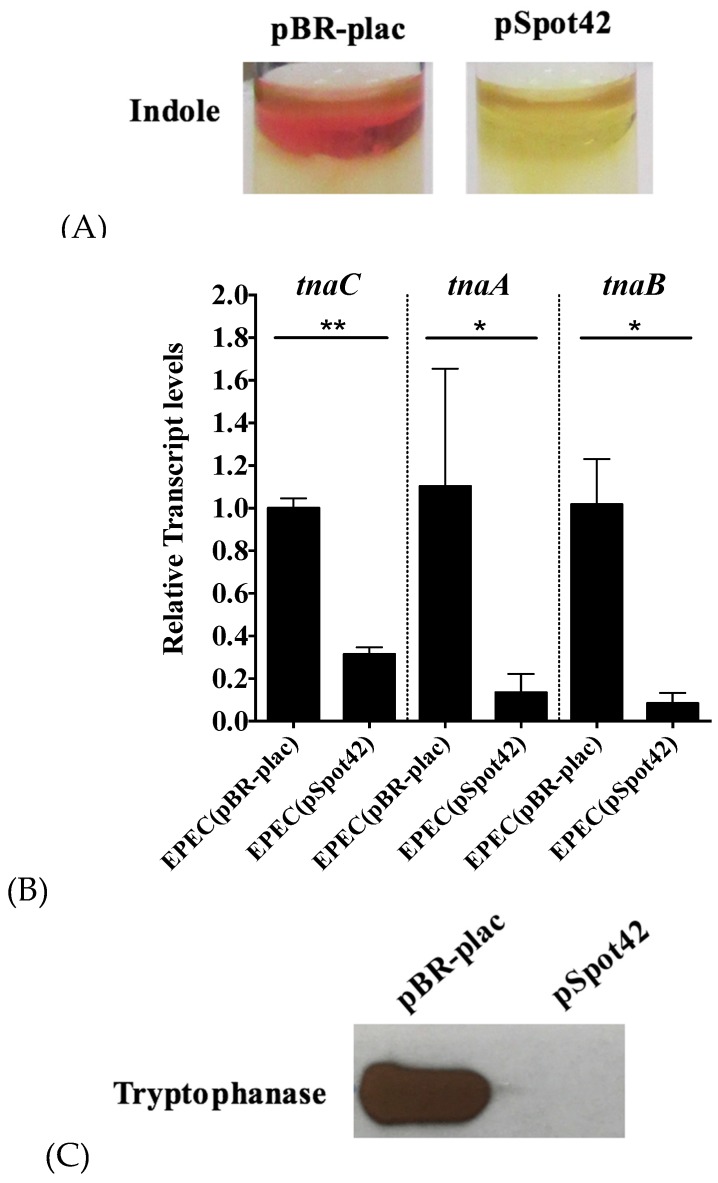
(**A**–**C**). Overproduction of Spot42 inhibits indole synthesis by repressing the *tnaCAB* mRNA. Overnight grown cultures of EPEC (pBR-plac) or EPEC (pSpot42) were sub-cultured in Luria-Bertani (LB) medium supplemented with ampicillin and IPTG and grown under static conditions to an OD_600_ of 1.2–1.4, after which Kovacs’ reagent was added to assay for indole production (**A**). For total RNA and protein extraction, overnight cultures of EPEC (pBR-plac) and EPEC (pSpot42) were sub-cultured identically, with the exception that cultures were grown under shaking conditions. The steady-state levels of *tnaC*, *tnaA*, and *tnaB* transcripts were determined by qRT-PCR and normalized with respect to the 16S rRNA transcript (**B**), whereas the tryptophanase protein level was quantified by western blotting (**C**). Each experiment was repeated on at least two separate occasions with replicates being used in each experiment. Similar results were obtained in each trial. The depicted results are representative of one such trial. For the qRT-PCR, error bars depict standard deviation. Student’s *t*-test was used to assay for statistical significance of the difference in the means between the wild type strain containing pBR-plac and the Spot42 overexpressor. A *p*-value of < 0.05 was considered to be statistically significant. ** denotes a *p*-value < 0.005 and * denotes a *p*-value of < 0.05. For Western blotting, protein loading was controlled for by assaying for total protein transfer using Ponceau S stain following electroblotting.

We used a genetic screen to identify sRNA regulators of indole biosynthesis as described in materials and methods. EPEC transformants were grown to the desired optical density and qualitatively assayed for indole synthesis by the addition of Kovacs reagent, which reacts with indole to produce the red colored pigment, rosindole [40]. Thus, the intensity of the red color directly correlates with the amount of indole biosynthesis. It was observed that, of all the sRNAs, Spot42 maximally inhibited indole biosynthesis, evident from the reduced synthesis of rosindole (Figure 1A). Next, we interrogated the molecular basis of the resulting phenotype. Consistent with the observed reduction in indole levels, overproduction of Spot42 negatively regulated the steady-state levels of *tnaC*, *tnaA*, and *tnaB* (Figure 1B). The observed reduction in transcript levels resulted in reduced protein expression as the abundance of tryptophanase was also reduced in the overproducer (Figure 1C). Collectively, these results suggest that overexpression of the Hfq-dependent sRNA Spot42 inhibits indole production by negatively regulating the transcript levels of *tnaCAB*. 

### 3.2. The tnaC-tnaA Intergenic Region Is Sufficient for Spot42-Dependent Repression

**Figure 2:**
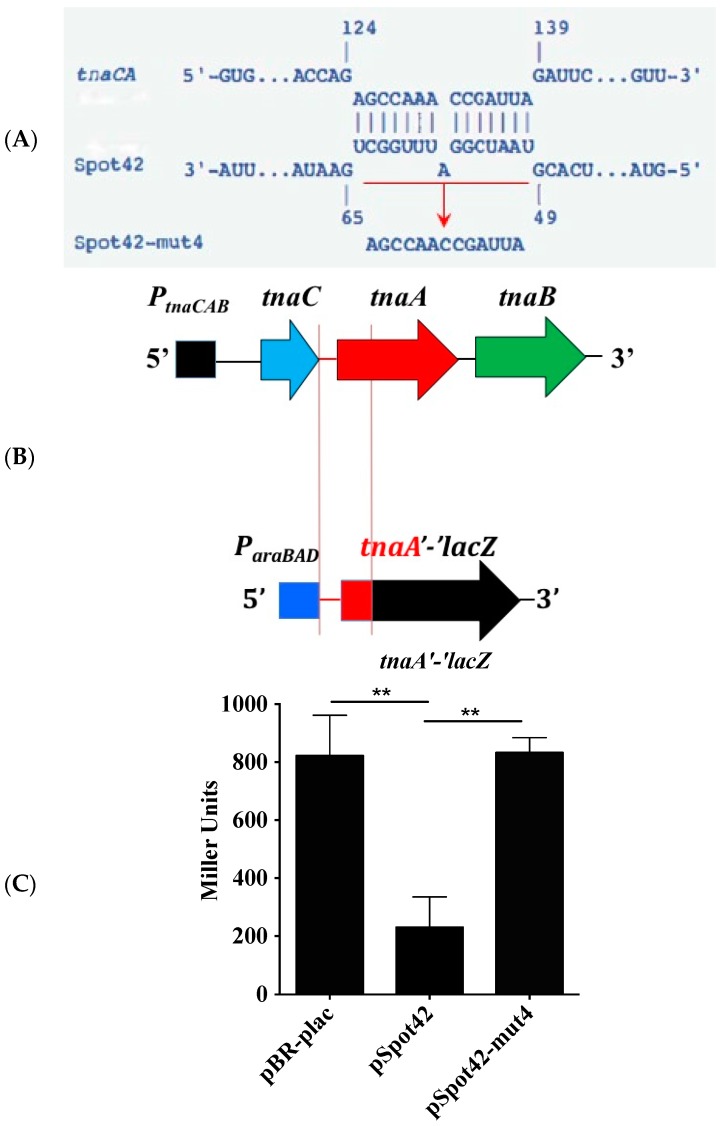
(**A**–**C**). Spot42 base-pairs to the *tnaCA* intergenic region to exert its silencing effect. IntaRNA revealed a region of complementarity between Spot42 and the intergenic region *tnaC-tnaA*, located immediately downstream of the Rho-dependent transcription terminator of *tnaC* (**A**). A polynucleotide substitution in Spot42, which generates the Spot42-mut4 allele, is predicted to abolish base-pairing and the ensuing negative regulation of *tnaCAB* by Spot42 (**A**). The *tnaCA* intergenic region along with 45 nucleotides of the *tnaA* ORF were fused to a truncated *lacZ* gene, which lacks its native 5′ UTR and 9 of the N-terminal codons, to generate a *tnaA’-‘lacZ* translational fusion. This fusion is transcriptionally driven by the heterologous *P_araBAD_* promoter (**B**). The *tnaA’-‘lacZ* reporter strains harboring pBR-plac or pSpot42 were grown in LB supplemented with ampicillin, arabinose, and IPTG. Overexpression of Spot42 repressed β-galactosidase activity from the minimal *P_araBAD_-tnaA’-‘lacZ* translational fusion and mutation of the predicted base-pairing region of *spf* prevented Spot42 from repressing the *tnaA’-‘lacZ* fusion (**C**). Each experiment was repeated on at least two separate occasions with replicates being used in each experiment. Similar results were obtained in each trial. The depicted results are representative of one such trial. Error bars depict standard deviation. Student’s *t*-test was used to assay for statistical significance of the difference in the means between EPEC(pBR-plac)/EPEC(pSpot42) and EPEC(pSpot42)/EPEC(pSpot42-mut4) pairs. A *p*-value of < 0.05 was considered to be statistically significant. ** denotes a *p*-value < 0.005.

Because Spot42, like the vast majority of Hfq-dependent sRNAs, exerts its effect by base-pairing to target mRNAs, next, we aligned the sRNA with the *tnaCAB* mRNA to identify potential regions of complementarity [41]. IntaRNA revealed a 15-nucleotide tract on Spot42 that was complementary to the intergenic region of *tnaC* and *tnaA*, situated downstream of the Rho-dependent transcription terminator of *tnaC* (Figure 2A) [32]. This suggests that Spot42 may directly base pair to the *tnaCAB* mRNA and affect mRNA stability and/or translation. Interestingly, the predicted base-pairing region of Spot42 has previously been shown to be important in antisense regulation of multiple mRNAs in *E. coli* [37]. To test for antisense regulation, the predicted intergenic region of *tnaC-tnaA* along with 45 nucleotides of the downstream *tnaA* open reading frame (ORF) were recombineered upstream of a truncated ‘*lacZ* gene that lacks its native 5′ UTR and translation initiation codon (Figure 2B). Recombineering generates a single copy *tnaA’-‘lacZ* translational fusion whereby the *lacZ* gene is under the posttranscriptional regulatory elements of *tnaA* and transcriptional control of the *araBAD* promoter (*P_araBAD_-tnaA’-‘lacZ*) (Figure 2B) [34]. Overexpression of Spot42 in this reporter *E. coli* strain diminished β-galactosidase activity from the minimal *P_araBAD_-tnaA’-‘lacZ* fusion by 4-fold (Figure 2C), while having no effect on the control *P_araBAD_-ler’-‘lacZ* fusion (data not shown). These results suggest that Spot42 specifically targets the 5′ leader region of *tnaA* to repress gene expression. To further validate duplex formation, we used a previously engineered Spot42 mutant allele, Spot42-II, containing a trinucleotide substitution in the predicted base-pairing motif [37]. However, this mutant allele was fully proficient in repressing the *tnaA’-‘lacZ* fusion (data not shown), suggesting that perhaps a larger polynucleotide mutation was necessary for any observable perturbation in regulation by Spot42. Therefore, we engineered a mutant allele, designated Spot42-mut4, in which the entire base-pairing region (15 nucleotides) was substituted with a scrambled sequence (Figure 2A). The Spot42-mut4 allele is not predicted to base pair to the wild type *tnaA’-‘lacZ* fusion. Consistent with this prediction, Spot42-mut4 did not repress β-galactosidase activity from the *P_araBAD_-tnaA’-‘lacZ* fusion (Figure 2C), confirming the essentiality of the polynucleotide tract in antisense regulation of the *tnaA’-‘lacZ* translational fusion by Spot42.

### 3.3. Spot42 Represses Transcription from the LEE1 Promoter

**Figure 3:**
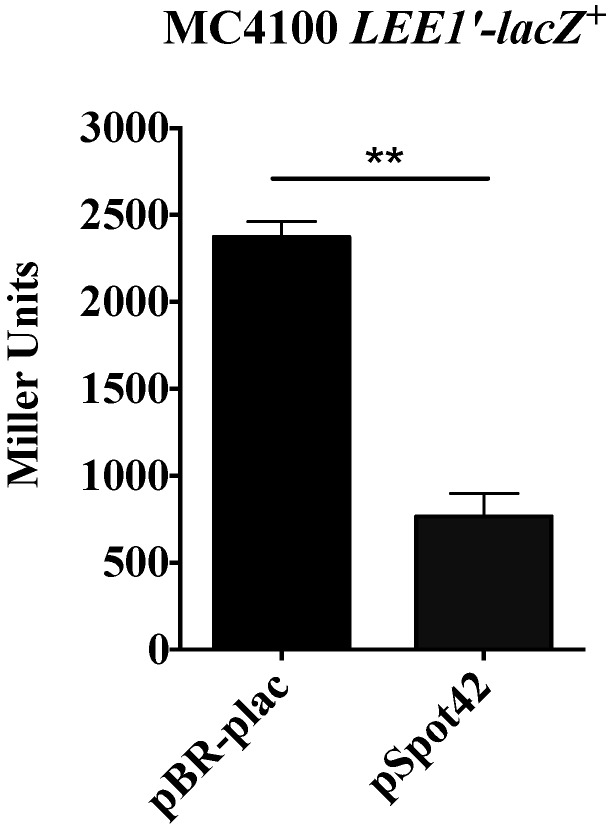
Spot42 represses transcription of *ler* and Ler-regulated targets. *E. coli* strain MC4100 *LEE1’-lacZ*^+^ transformants containing pBR-plac or pSpot42 were grown in LB broth containing ampicillin and IPTG to an OD_600_ of 1.1–1.4 and assayed for β-galactosidase activity. Overexpression of Spot42 repressed β-galactosidase activity from the *LEE1’-lacZ*^+^ fusion. Each assay was conducted on at least two separate occasions with replicate samples being used in each trial. Student’s *t*-test was used to assay for statistical significance of the difference in the means between the *LEE1’-lacZ*^+^ (pBR-plac) vs. *LEE1’-lacZ*^+^ (pSpot42). A *p*-value of < 0.05 was considered to be statistically significant. ** denotes a *p*-value < 0.005.

The metabolite indole activates transcription from the *LEE1* promoter in EPEC [31]. The first gene in the *LEE1* operon encodes Ler, which is the master regulator that spatiotemporally synchronizes transcription from the other *LEE* operons (*LEE2–5*) to stimulate morphogenesis of A/E lesions. Because indole levels were down-regulated in the Spot42 overproducer, we hypothesized that transcriptional activity from *LEE1* would be diminished. Consistent with this prediction, overexpression of Spot42 negatively regulated β-galactosidase activity by 3-fold in a reporter *E. coli* strain harboring a chromosomal *P_LEE1_-ler’-lacZ*^+^ transcriptional fusion (Figure 3). Because transcription from the *LEE1* promoter is reduced, one would anticipate a correlative reduction in expression of Ler-activated targets and A/E lesion biogenesis.

**Figure 4:**
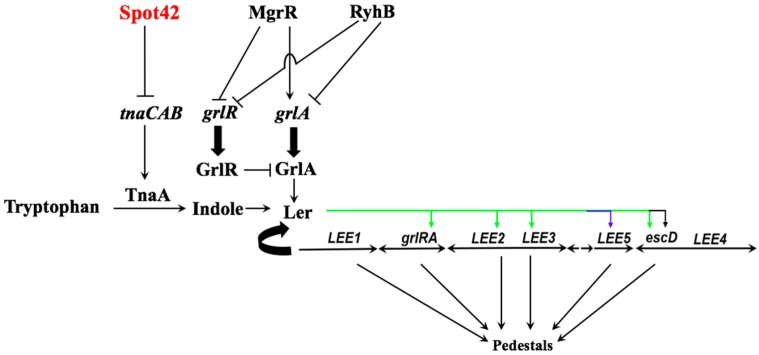
Model for the Spot42-dependent regulation of the LEE via indole biosynthesis. Spot42 base-pairs to the *tnaCAB* mRNA and presumably destabilizes the entire transcript. Reduced synthesis of TnaB (tryptophan permease) and TnaA (tryptophanase) results in reduced import and hydrolysis of tryptophan to indole. The reduced intracellular levels of indole, in turn, would result in reduced transcriptional activation from the *LEE1* promoter of EPEC. Reduced expression of the *LEE1*-encoded master regulator Ler, in turn, would lead to reduced gene expression from the other LEE operons. Thus, in EPEC, overexpression of Spot42 negatively regulates indole biosynthesis to diminish gene expression from the LEE.

In summary, we have demonstrated that the Hfq-dependent sRNA Spot42 base-pairs to the *tnaCAB* mRNA to globally silence gene expression from the entire transcript presumably by destabilizing it. The reduction in mRNA levels, in turn, leads to reduced expression and steady-state levels of the structural and regulatory proteins involved in indole biosynthesis (Figure 4). In the absence of indole, the *LEE1* promoter is not optimally active and Ler transcription is reduced (Figure 4), which, in turn, is expected to delay and diminish the morphogenesis of A/E lesion. 

The nutritional status of a bacterium can impact its virulence potential. For instance, the quality and/or quantity of a carbon source, such as sugars, dictate the molecular epidemiology of a bacterial infection [42,43]. Reciprocally, prominent metabolic regulators, such as CRP, Cra, KdpE, and CsrA, that perceive environmental carbon sources and/or monitor the intracellular metabolic state, moonlight as virulence regulators in diverse pathogens [25,44,45]. The catabolite-responsive Hfq-dependent sRNA Spot42, specified by the *spf* gene, is a well-studied riboregulator that is present in both pathogenic and nonpathogenic strains of *E. coli* [37,46]. The sRNA enables its host bacterium to switch between different carbon sources [37,47]. Preferred carbon sources, such as glucose, induce transcription of Spot42 in a CRP-dependent manner [48]. Spot42, in turn, base-pairs to transcripts to repress the expression of proteins involved in the uptake and/or metabolism of secondary carbon sources, thereby enabling the bacterium to utilize the preferred carbon source [37]. Upon depletion of the preferred carbon source, cAMP levels are elevated, which, in turn, interacts with CRP and activates it [49,50]. The CRP-cAMP holoprotein represses transcription of *spf*, thereby derepressing mRNAs involved in metabolism of secondary carbon sources [37,48]. Thus, CRP and Spot42 are vital regulators involved in carbon catabolite repression (CCR) [51]. Whereas CRP is the primary transcriptional factor involved in CCR in *E. coli* [51], Spot42 fine-tunes CCR by posttranscriptional control of the synthesized mRNAs [37,52].

The *tnaCAB* catabolic operon houses the regulatory and structural gene products that enables its host bacterium to utilize tryptophan as a carbon and energy source [28,53]. Additionally, in EPEC, the hydrolytic product of tryptophan, indole, also induces transcription from the *LEE1* promoter, thereby affecting bacterial virulence. Thus, the gene products encoded within the *tnaCAB* operon bridge cellular metabolism with virulence in EPEC. As expected, in *E. coli*, *tnaCAB* is transcriptionally activated by CRP [54,55]. The promoter architecture of the *tnaCAB* operon is preserved between *E. coli* and EPEC, suggesting that the CRP-dependent transcriptional regulation is intact in this A/E pathogen [25]. Our discovery that the polycistronic *tnaCAB* mRNA is also subject to antisense regulation by Spot42 suggests that CCR exerts a multi-tiered regulatory control to fine-tune gene expression from the *tnaCAB* operon. Based on our results, it is reasonable to hypothesize that Spot42 levels are likely to be higher in EPEC when the bacterium is in an environment where the expression from the LEE is not needed, such as outside of the mammalian host. Upon entry into the small intestine, Spot42 levels are likely to be down-regulated. This down-regulation would lead to derepression of the *tnaCAB* mRNA and stimulate indole biosynthesis. Indole production, in turn, would activate *ler* and prime the LEE regulatory cascade to culminate with the biogenesis of A/E lesions. Experiments to test this hypothesis are underway in our lab. 

In summary, the presented results expand the repertoire of genes, co-regulated by both CRP and Spot42, that bridge bacterial metabolism and virulence. Moreover, these results also highlight a recurring, but underappreciated, theme in the growing body of work on A/E pathogens, about the importance of regulatory RNAs in modulating pathogenetic pathways. 

## Figures and Tables

**Table 1:** Bacterial strains and plasmids used in this study.

Strain	Relevant Genotype	Reference or Source
LS4923	EPEC strain E2348/69 (pBR-plac), Amp^R^	This study
LS4942	E2348/69 (pSpot42) Transformant #1, Amp^R^	This study
LS4943	E2348/69 (pSpot42) Transformant #2, Amp^R^	This study
LS4767 = PM1205	*P_araBAD_-cat-sacB-‘lacZ* mini-lambda, Cm^R^ Tet^R^ Suc^S^	[34]
LS5454	LS4767 *P_araBAD_-tnaA’-‘lacZ* Recombinant #1, Cm^S^ Tet^S^ Suc^R^	This study
LS5455	LS4767 *P_araBAD_-tnaA’-‘lacZ* Recombinant #2, Cm^S^ Tet^S^ Suc^R^	This study
LS5457	LS5454 (pBR-plac) Transformant #1, Cm^S^ Tet^S^ Suc^R^ Amp^R^	This study
LS5462	LS5455 (pBR-plac) Transformant #2, Cm^S^ Tet^S^ Suc^R^ Amp^R^	This study
LS5459	LS5454 (pSpot42) Transformant #1, Cm^S^ Tet^S^ Suc^R^ Amp^R^	This study
LS5463	LS5455 (pSpot42) Transformant #2, Cm^S^ Tet^S^ Suc^R^ Amp^R^	This study
LS5667	LS5454 (pSpot42-mut4) Transformant #1, Cm^S^ Tet^S^ Suc^R^ Amp^R^	This study
LS5670	LS5455 (pSpot42-mut4) Transformant #2, Cm^S^ Tet^S^ Suc^R^ Amp^R^	This study
JLM164 = LS4052	MC4100 *LEE1’-lacZ^+^*	[21]
LS5698	LS4052 (pBR-plac) Transformant #1, Amp^R^	This study
LS5699	LS4052 (pBR-plac) Transformant #2, Amp^R^	This study
LS5710	LS4052 (pSpot42) Transformant #1, Amp^R^	This study
LS5711	LS4052 (pSpot42) Transformant #2, Amp^R^	This study
DH5α	*supE44 ∆lacU169* (Ф80 *lacZ∆*M15) *hsdR17 recA1 endA1 gryA96 thi-1 relA1*	[35]
Plasmids		
pBR-plac	Cloning vector, Amp^R^	[36]
pSpot42	*spf* wild type allele under an IPTG inducible promoter, Amp^R^	[37]
pSpot42-mut4	*spf_mut4_* allele under an IPTG inducible promoter, Amp^R^	This study

Cm^R/S^—chloramphenicol resistant/sensitive, Str^R/S^—streptomycin resistant/sensitive, Amp^R/S^—ampicillin resistant/sensitive, Suc^R/S^—Sucroses resistant/sensitive.

**Table 2:** Oligonucleotides used in this study.

Primers (Purpose)	Sequence
SB2414 (5′ primer for generating *P_araBAD_-tnaA’-‘lacZ* fusion)	ACCTGACGCTTTTTATCGCAACTCTCTACTGTTTCTCCATccttctgtagccatcaccag
SB2415 (3′ primer for generating *P_araBAD_-tnaA’-‘lacZ* fusion)	TAACGCCAGGGTTTTCCCAGTCACGACGTTGTAAAACGACaacacgaatgcggaacggtt
SB2181 (5′ primer for sequencing *P_araBAD_-tnaA’-‘lacZ* fusion)	CGACGAATTCGCGCTTCAGCCATACTTTTCATAC
SB2180 (3′ primer for sequencing *P_araBAD_-tnaA’-‘lacZ* fusion)	CGGGCCTCTTCGCTA
SB2458b (5′ primer to generate the *spf_mut4_* allele in pBR-plac)	CTTTCAGACCTTTTACTTCACGattagccaaccgaGAATATTTTAGCCGCCCCAGTC
SB2459 (3′ primer to generate the *spf_mut4_* allele in pBR-plac)	ATAGAACATCTTACCTCTGTACCCT
SB2323 (5′ primer to confirm *spf_mut4_* allele in pSpot42-mut4)	GCGACACGGAAATGTTGAATAC
SB2324 (3′ primer to confirm *spf_mut4_* allele in pSpot42-mut4)	CAGTACCGGCATAACCAAGC
5′ *tnaC* (upstream primer for qRT-PCR)	ATGAATATCTTACATATATGTGTGACCTCA
3′ *tnaC* (downstream primer qRT-PCR)	CAAGGGCGGTGATCGACAATC
5′ *tnaA* (upstream primer for qRT-PCR)	AGCAGCGTGAAGCAGAATACA
3′ *tnaA* (downstream primer for qRT-PCR)	TGACTCGGCTAACGCATAGTAGC
5′ *tnaB* (upstream primer for qRT-PCR)	CGGTAACACCTGGAACATTATCAGC
3′ *tnaB* (downstream primer for qRT-PCR)	AATGATCGCACCATTAGCAGAG
5′ 16S rRNA (upstream primer for qRT-PCR)	CTTACGACCAGGGCTACACAC
3’ 16S rRNA (downstream primer for qRT-PCR)	CGGACTACGACGCACTTTATG

## References

[1] Waters L.S., Storz G. (2009). Regulatory RNAs in bacteria. Cell.

[2] Papenfort K., Vogel J. (2010). Regulatory RNA in bacterial pathogens. Cell Host Microbe.

[3] Beisel C.L., Storz G. (2010). Base pairing small RNAs and their roles in global regulatory networks. FEMS Microbiol. Rev..

[4] Bhatt S., Egan M., Jenkins V., Muche S., El-Fenej J. (2016). The tip of the iceberg: On the roles of regulatory small RNAs in the virulence of enterohemorrhagic and enteropathogenic *Escherichia coli*. Front. Cell Infect. Microbiol..

[5] Link T.M., Valentin-Hansen P., Brennan R.G. (2009). Structure of *Escherichia coli* Hfq bound to polyriboadenylate RNA. Proc. Natl. Acad. Sci. USA.

[6] Chao Y., Vogel J. (2010). The role of Hfq in bacterial pathogens. Curr. Opin. Microbiol..

[7] Tree J.J., Granneman S., McAteer S.P., Tollervey D., Gally D.L. (2014). Identification of bacteriophage-encoded anti-sRNAs in pathogenic *Escherichia coli*. Mol. Cell.

[8] Sittka A., Pfeiffer V., Tedin K., Vogel J. (2007). The RNA chaperone Hfq is essential for the virulence of *Salmonella Typhimurium*. Mol. Microbiol..

[9] Gruber C.C., Sperandio V. (2015). Global analysis of post-transcriptional regulation by glmy and glmz in enterohemorrhagic *E. coli* (EHEC) o157:H7. Infect. Immun..

[10] Robins-Browne R.M. (1987). Traditional enteropathogenic *Escherichia coli* of infantile diarrhea. Rev. Infect. Dis..

[11] Mellies J.L., Barron A.M., Carmona A.M. (2007). Enteropathogenic and enterohemorrhagic *Escherichia coli* virulence gene regulation. Infect. Immun..

[12] Moon H.W., Whipp S.C., Argenzio R.A., Levine M.M., Giannella R.A. (1983). Attaching and effacing activities of rabbit and human enteropathogenic *Escherichia coli* in pig and rabbit intestines. Infect. Immun..

[13] Bhatt S., Romeo T., Kalman D. (2011). Honing the message: Post-transcriptional and post-translational control in attaching and effacing pathogens. Trends Microbiol..

[14] Kenny B., DeVinney R., Stein M., Reinscheid D.J., Frey E.A., Finlay B.B. (1997). Enteropathogenic *E. coli* (EPEC) transfers its receptor for intimate adherence into mammalian cells. Cell.

[15] Knutton S., Baldwin T., Williams P.H., McNeish A.S. (1989). Actin accumulation at sites of bacterial adhesion to tissue culture cells: Basis of a new diagnostic test for enteropathogenic and enterohemorrhagic *Escherichia coli*. Infect. Immun..

[16] McDaniel T.K., Kaper J.B. (1997). A cloned pathogenicity island from enteropathogenic *Escherichia coli* confers the attaching and effacing phenotype on *E. Coli* K-12. Mol. Microbiol..

[17] Jarvis K.G., Giron J.A., Jerse A.E., McDaniel T.K., Donnenberg M.S., Kaper J.B. (1995). Enteropathogenic *Escherichia coli* contains a putative type III secretion system necessary for the export of proteins involved in attaching and effacing lesion formation. Proc. Natl. Acad. Sci. USA.

[18] Knutton S., Rosenshine I., Pallen M.J., Nisan I., Neves B.C., Bain C., Wolff C., Dougan G., Frankel G. (1998). A novel espa-associated surface organelle of enteropathogenic *Escherichia coli* involved in protein translocation into epithelial cells. EMBO J..

[19] Bhatt S., Egan M., Ramirez J., Xander C., Jenkins V., Muche S., El-Fenej J., Palmer J., Mason E., Storm E. (2017). Hfq and three Hfq-dependent small regulatory RNAs-MgrR, RyhB and McaS-coregulate the locus of enterocyte effacement in enteropathogenic *Escherichia coli*. Pathog. Dis..

[20] Deng W., Puente J.L., Gruenheid S., Li Y., Vallance B.A., Vazquez A., Barba J., Ibarra J.A., O’Donnell P., Metalnikov P. (2004). Dissecting virulence: Systematic and functional analyses of a pathogenicity island. Proc. Natl. Acad. Sci. USA.

[21] Mellies J.L., Elliott S.J., Sperandio V., Donnenberg M.S., Kaper J.B. (1999). The Per regulon of enteropathogenic *Escherichia coli*: Identification of a regulatory cascade and a novel transcriptional activator, the locus of enterocyte effacement (LEE)-encoded regulator (Ler). Mol. Microbiol..

[22] Huang L.H., Syu W.J. (2008). GrlA of enterohemorrhagic *Escherichia coli* O157:H7 activates LEE1 by binding to the promoter region. J. Microbiol. Immunol. Infect..

[23] Jobichen C., Li M., Yerushalmi G., Tan Y.W., Mok Y.K., Rosenshine I., Leung K.Y., Sivaraman J. (2007). Structure of GrlR and the implication of its EDED motif in mediating the regulation of type III secretion system in EHEC. PLoS Pathog..

[24] Yanofsky C., Konan K.V., Sarsero J.P. (1996). Some novel transcription attenuation mechanisms used by bacteria. Biochimie.

[25] Bhatt S., Anyanful A., Kalman D. (2011). CsrA and TnaB coregulate tryptophanase activity to promote exotoxin-induced killing of *Caenorhabditis elegans* by enteropathogenic *Escherichia coli*. J. Bacteriol..

[26] Ng H., Gartner T.K. (1963). Selection of mutants of *Escherichia coli* constitutive for tryptophanase. J. Bacteriol..

[27] Gong F., Ito K., Nakamura Y., Yanofsky C. (2001). The mechanism of tryptophan induction of tryptophanase operon expression: Tryptophan inhibits release factor-mediated cleavage of TnaC-peptidyl-tRNA(Pro). Proc. Natl. Acad. Sci. USA.

[28] Deeley M.C., Yanofsky C. (1981). Nucleotide sequence of the structural gene for tryptophanase of *Escherichia coli* K-12. J. Bacteriol..

[29] Yanofsky C., Horn V., Gollnick P. (1991). Physiological studies of tryptophan transport and tryptophanase operon induction in *Escherichia coli*. J. Bacteriol..

[30] Wood W.A., Gunsalus I.C., Umbreit W.W. (1947). Pyridoxal phosphate as the coenzyme of tryptophanase from *Escherichia coli*. J. Bacteriol..

[31] Anyanful A., Dolan-Livengood J.M., Lewis T., Sheth S., Dezalia M.N., Sherman M.A., Kalman L.V., Benian G.M., Kalman D. (2005). Paralysis and killing of *Caenorhabditis elegans* by enteropathogenic *Escherichia coli* requires the bacterial tryptophanase gene. Mol. Microbiol..

[32] Konan K.V., Yanofsky C. (2000). Rho-dependent transcription termination in the *tna* operon of *Escherichia coli*: Roles of the boxa sequence and the rut site. J. Bacteriol..

[33] Gong F., Yanofsky C. (2001). Reproducing *tna* operon regulation in vitro in an S-30 system. Tryptophan induction inhibits cleavage of TnaC peptidyl-tRNA. J. Biol. Chem..

[34] Mandin P., Gottesman S. (2009). A genetic approach for finding small RNAs regulators of genes of interest identifies RybC as regulating the DpiA/DpiB two-component system. Mol. Microbiol..

[35] Taylor R.G., Walker D.C., McInnes R.R. (1993). *E. coli* host strains significantly affect the quality of small scale plasmid DNA preparations used for sequencing. Nucleic Acids Res..

[36] Guillier M., Gottesman S. (2006). Remodelling of the *Escherichia coli* outer membrane by two small regulatory rnas. Mol. Microbiol..

[37] Beisel C.L., Storz G. (2011). The base-pairing RNA spot 42 participates in a multioutput feedforward loop to help enact catabolite repression in *Escherichia coli*. Mol. Cell.

[38] Sambrook J., Russell D.W. (2001). Molecular Cloning: A Laboratory Manual.

[39] Mandin P., Gottesman S. (2010). Integrating anaerobic/aerobic sensing and the general stress response through the ArcZ small RNA. EMBO J..

[40] McFaddin J.F. (1980). Biochemical Tests for Identification of Medical Bacteria.

[41] Busch A., Richter A.S., Backofen R. (2008). Intarna: Efficient prediction of bacterial sRNA targets incorporating target site accessibility and seed regions. Bioinformatics.

[42] Le Bouguenec C., Schouler C. (2011). Sugar metabolism, an additional virulence factor in enterobacteria. Int. J. Med. Microbiol..

[43] Baumler A.J., Sperandio V. (2016). Interactions between the microbiota and pathogenic bacteria in the gut. Nature.

[44] Njoroge J.W., Gruber C., Sperandio V. (2013). The interacting Cra and KdpE regulators are involved in the expression of multiple virulence factors in enterohemorrhagic *Escherichia coli*. J. Bacteriol..

[45] Bhatt S., Edwards A.N., Nguyen H.T., Merlin D., Romeo T., Kalman D. (2009). The RNA binding protein CsrA is a pleiotropic regulator of the locus of enterocyte effacement pathogenicity island of enteropathogenic *Escherichia coli*. Infect. Immun..

[46] Papenfort K., Vogel J. (2011). Sweet business: Spot42 RNA networks with CRP to modulate catabolite repression. Mol. Cell.

[47] Moller T., Franch T., Udesen C., Gerdes K., Valentin-Hansen P. (2002). Spot42 RNA mediates discoordinate expression of the *E. coli* galactose operon. Genes Dev..

[48] Polayes D.A., Rice P.W., Garner M.M., Dahlberg J.E. (1988). Cyclic AMP-cyclic AMP receptor protein as a repressor of transcription of the spf gene of *Escherichia coli*. J. Bacteriol..

[49] Zubay G., Schwartz D., Beckwith J. (1970). Mechanism of activation of catabolite-sensitive genes: A positive control system. Proc. Natl. Acad. Sci. USA.

[50] Emmer M., deCrombrugghe B., Pastan I., Perlman R. (1970). Cyclic amp receptor protein of *E. coli*: Its role in the synthesis of inducible enzymes. Proc. Natl. Acad. Sci. USA.

[51] Gorke B., Stulke J. (2008). Carbon catabolite repression in bacteria: Many ways to make the most out of nutrients. Nat. Rev. Microbiol..

[52] Gorke B., Vogel J. (2008). Noncoding RNA control of the making and breaking of sugars. Genes Dev..

[53] Edwards R.M., Yudkin M.D. (1982). Location of the gene for the low-affinity tryptophan-specific permease of *Escherichia coli*. Biochem. J..

[54] Isaacs H., Chao D., Yanofsky C., Saier M.H. (1994). Mechanism of catabolite repression of tryptophanase synthesis in *Escherichia coli*. Microbiology.

[55] Deeley M.C., Yanofsky C. (1982). Transcription initiation at the tryptophanase promoter of *Escherichia coli* K-12. J. Bacteriol..

